# Lectins from *Synadenium carinatum* (ScLL) and *Artocarpus heterophyllus* (ArtinM) Are Able to Induce Beneficial Immunomodulatory Effects in a Murine Model for Treatment of *Toxoplasma gondii* Infection

**DOI:** 10.3389/fcimb.2016.00164

**Published:** 2016-11-25

**Authors:** Leandro Peixoto Ferreira de Souza, Eliézer L. P. Ramos, Silas S. Santana, Murilo V. Silva, Fernanda M. Santiago, Tiago W. P. Mineo, José R. Mineo

**Affiliations:** Laboratório de Imunoparasitologia, Instituto de Ciências Biomédicas—Universidade Federal de UberlândiaUberlândia, Brazil

**Keywords:** *Toxoplasma gondii*, lectins, ScLL, ArtinM, therapeutic agents

## Abstract

Infection by *Toxoplasma gondii* affects around one-third of world population and the treatment for patients presenting toxoplasmosis clinically manifested disease is mainly based by a combination of sulfadiazine, pyrimethamine, and folinic acid. However, this therapeutic protocol is significantly toxic, causing relevant dose-related bone marrow damage. Thus, it is necessary to improve new approaches to investigate the usefulness of more effective and non-toxic agents for treatment of patients with toxoplasmosis. It has been described that lectins from plants can control parasite infections, when used as immunological adjuvants in vaccination procedures. This type of lectins, such as ArtinM and ScLL is able to induce immunostimulatory activities, including efficient immune response against parasites. The present study aimed to evaluate the potential immunostimulatory effect of ScLL and ArtinM for treatment of *T. gondii* infection during acute phase, considering that there is no study in the literature accomplishing this issue. For this purpose, bone marrow-derived macrophages (BMDMs) were treated with different concentrations from each lectin to determine the maximum concentration without or with lowest cytotoxic effect. After, it was also measured the cytokine levels produced by these cells when stimulated by the selected concentrations of lectins. We found that ScLL showed high capacity to induce of pro-inflammatory cytokine production, while ArtinM was able to induce especially an anti-inflammatory cytokines production. Furthermore, both lectins were able to increase NO levels. Next, we evaluated the treatment effect of ScLL and ArtinM in C57BL/6 mice infected by ME49 strain from *T. gondii*. The animals were infected and treated with ScLL, ArtinM, ArtinM plus ScLL, or sulfadiazine, and the following parameters analyzed: Cytokines production, brain parasite burden and survival rates. Our results demonstrated that the ScLL or ScLL plus ArtinM treatment induced production of pro-inflammatory and anti-inflammatory cytokines, showing differential but complementary profiles. Moreover, when compared with non-treated mice, the parasite burden was significantly lower and survival rates higher in mice treated with ScLL or ScLL plus ArtinM, similarly with sulfadiazine treatment. In conclusion, the results demonstrated the suitable potential immunotherapeutic effect of ScLL and ArtinM lectins to control acute toxoplasmosis in this experimental murine model.

## Introduction

*Toxoplasma gondii* is an obligate intracellular apicomplexan parasite, and it is the etiologic agent of toxoplasmosis, being able to infect virtually all warm blood vertebrates, including human beings (Dubey et al., 1998, 2012; Tenter et al., 2000; Samra et al., 2007; Lopes et al., 2014).

This infection is asymptomatic and well tolerated for the majority of the infected people, but it can cause severe disease and high rates of morbidity and mortality for some groups of patients, as the immunocompromised individuals, such as for AIDS patients (Enzensberger et al., 1985; Bal et al., 2014), as well as when it occurs during pregnancy, because the parasite can cross placenta and cause congenital toxoplasmosis (Jones et al., 2001; Adams Waldorf and McAdams, 2013). Thus, the treatment of toxoplasmosis is required for these patients presenting high risk of severe tissue damage (Vijayalaxmi and Vishalakshi, 2000; Montoya and Liesenfeld, 2004; Elsheikha, 2008; Kaye, 2011; Rodriguez and Szajnman, 2012; Blader et al., 2015). If fetal infection is confirmed, the mother should be treated with a combination of sulfadiazine, pyrimethamine, and folinic acid (Montoya and Remington, 2008). Even though sulfadiazine and pyrimethamine are widely used, these drugs are highly toxic and may cause severe adverse effects (Montoya and Remington, 2008; Kaye, 2011). In fact, these drugs may result in bone marrow toxicity, including megaloblastic anemia or pancytopenia, which may be reversible or preventable in some patients with folate supplementation (Mori et al., 2011). In addition to cause these severe side effects, these drugs might not be able to reduce the parasitism, as *T. gondii* has shown to present resistance to sulfadiazine (Meneceur et al., 2008; Doliwa et al., 2013; Oliveira et al., 2016).

The immune response against *T. gondii* involves complex mechanisms of innate and adaptive immunity. A Th1-type immune response is observed during acute infection, involving synthesis of cytokines, as IFN-γ and IL-12 (Gazzinelli et al., 1994; Lang et al., 2007). Given that modulated immunity is critical to control the parasite burden (Dupont et al., 2012), the induction of an appropriate immune response just after infection constitutes an remarkable alternative for toxoplasmosis treatment.

It has been described in the literature that lectins from plants, such as ArtinM from seeds of jackfruit (*Artocarpus integrifolia*) and ScLL from the latex of the Euphorbiaceae *Synadenium carinatum*, when used as immunological adjuvants in vaccination protocols, can control parasite infections caused by *Leishmania major, Leishmania amazonensis* or *Neospora caninnun* (Panunto-Castelo et al., 2001; Teixeira et al., 2006; Afonso-Cardoso et al., 2007; Toledo et al., 2009; Cardoso et al., 2011).

Considering that it is necessary to improve new approaches to investigate the usefulness of more effective and non-toxic agents for treatment of patients with toxoplasmosis, in addition to the fact that ScLL and ArtinM have been previously used only in vaccination protocols for parasitic infections, the major aim of the present study was to evaluate whether these lectins could be also applicable as therapeutic agents to avoid the tissue damages occurring in consequence of *T. gondii* infection.

## Materials and methods

### Animals

Female inbred C57BL/6 mice, aging 8–10 weeks, were obtained from Federal University of Uberlândia (UFU), Uberlândia, MG, Brazil. Animals were maintained under standard conditions in the Animal Facility from this Institution. All procedures were conducted in accordance with the guidelines for animal ethics and the study received approval of the Ethics Committee for Animal Experimentation of the Institution (CEUA-UFU), under protocol # 058/14.

### Parasites

Brain cysts from ME49 strain of *T. gondii* were obtained from *Calomys callosus*, as previously described (Ferro et al., 2002). Briefly, animals were infected for 45 days and their brains were removed, washed in PBS and homogenized. *T. gondii* cyst numbers were counted by light microscopy and volumes of 200 μL of PBS containing 10 cysts were administered to mice to carry out the experimental procedures.

### Lectins from *Synadenium carinatum* latex (ScLL) and *Artocarpus heterophyllus* seeds (ArtinM)

Specimens from *S. carinatum* were harvested in Uberlândia, MG, Brazil. ScLL was obtained as previously described (Souza et al., 2005), with some modifications. Briefly, proteins were extracted from the fresh plant latex by gentle shaking with distilled water, for 24 h, at 4°C. The mixture was centrifuged for 10 min at 10,000 g, filtered on 0.45 μm membranes and frozen at −20°C for 48 h. After being defrosted and filtered again, the extract was submitted to affinity chromatography using immobilized D-galactose column on agarose (Pierce, Rockford, USA). The D-galactose-binding lectin (ScLL) was eluted with 0.2 M D-galactose (Sigma Chemical Co., St Louis, USA) in BBS, concentrated, and dialyzed using Amicon® Ultra-Filters 40 kDa (Merck, Göttingen, Germany). The protein concentration was determined (Bradford, 1976) and ScLL purity was confirmed by sodium dodecyl sulfate polyacrylamide gel electrophoresis (SDS-PAGE) stained with Coomassie Blue (Laemmli, 1970). ScLL aliquots were stored at −20°C until be used in the experimental procedures.

The D-mannose-binding ArtinM lectin extracted from *A. heterophyllus* was obtained from seeds of jackfruit and purified by sugar affinity chromatography, as previously described (Bunn-Moreno and Campos-Neto, 1981; Roque-Barreira et al., 1986).

### *In vitro* assays

#### Bone marrow-derived macrophages (BMDMs)

Bone marrow-derived macrophages (BMDMs) were obtained from C57BL/6 mice as previously described (Marim et al., 2010). Briefly, the femurs from mice (*n* = 2) were removed and washed with sterile PBS in Petri dish. The cell suspensions were collected, filtered, and centrifuged for 10 min at 400 g. The pellets were suspended in RPMI-1640 media containing 15% of LCCM (L-cell Conditioned Media) from L929 cell culture and incubated for 24 h. Cells were washed again and suspended in medium containing 2% SBF (serum bovine fetal). Viable cells were counted in a Neubauer chamber, using the Trypan blue exclusion vital stain.

#### Cytotoxicity assay—(MTT)

To assess the cytotoxic effect on macrophages treated with different concentrations of ScLL or ArtinM lectins, we performed the tetrazolium salt (MTT) colorimetric assay (Mosmann, 1983). Cells were treated with ScLL (50.0; 16.6; 5.5; 1.8; 0.61; 0.20; and 0.06 μg/mL) or ArtinM (1.0; 0.33; 0.11; 0.037; 0.012; 0.004; 0.00013; and 0.00004 μg/mL) and incubated at 37°C and 5% CO_2_ for 24 h before being analyzed.

#### Cytokines (IL-10, IL-12p40) and NO measurements

BMDMs were cultured and stimulated with ScLL (50.0, 16.6, 5.5, 1.8, 0.61, 0.20, and 0.06 μg/mL) or ArtinM (1.0, 0.33, 0.11 μg/mL). Cells stimulated by LPS (1 μg/mL) or medium alone were included in all experiments, as positive and negative controls, respectively. Cell supernatants were collected and stored at –70°C for detection of IL-10, IL-12p40 and nitrite. Cytokine measurements were performed by sandwich ELISA according to manufacturer's instructions (R&D Systems, Minneapolis, MN). The limit of detection was 31 pg/mL for all cytokines. Nitrite was measured by Griess Reaction (Green et al., 1982).

### Experimental infection and treatment

Mice (*n* = 45) were orally infected with 10 cysts of *T. gondii* ME49 strain and treated with sulfadiazine or lectins. For survival analysis, 25 animals were used, while 20 animals were used for cytokine measurement levels. Thirty days after infection, the surviving animals from both experiments were euthanized and their brains collected for determination of the parasite burden by Real-time PCR.

#### Survival analysis

Five groups (*n* = 5) of infected and treated mice were chosen for survival analysis. One group was treated with sulfadiazine (250 mg diluted in 400 mL of drinking water given to the animals for 6 days), whereas four groups were treated for 6 days, at 1-day intervals, intraperitoneally, according with the following preparations: 50 μg of ScLL; 50 μg of ScLL plus 1 μg of ArtinM; 1 μg ArtinM; or PBS only. These lectin concentrations were chosen based on previous studies (Cardoso et al., 2011, 2012). Thirty days after infection the surviving animals were euthanized and their brains collected to determine the rates of parasitism burden.

#### Measurement of serum levels of cytokines

The production of cytokines was assessed in blood samples from infected and treated mice, as well as before treatment and infection and at the end of the treatments, i.e., 7 days after the infection. The serum samples were stored at −70°C until being analyzed for measurement of IL-2, IL-4, IL-6, IL-10, IL-17, IFN-γ, and TNF cytokines by Cytometric Bead Array (CBA) (BD Bioscience, San Jose, USA), according to the manufacturer's instructions. Samples were analyzed under BD flow cytometry (FACSCanto II; BD Company, San Diego, USA), and data were analyzed by using FlowJo data analysis software (FlowJo, LLC, Ashland, USA). The results were expressed as mean concentration pg/mL.

### Determination of parasite burden in the brain tissues

Parasite burden was determined in the brain tissues by quantitative real-time polymerase chain reaction (qPCR). Total DNA was extracted using Proteinase K (Promega Co., Madison, WI, USA), as described by Miller et al. (1988). Total DNA was quantified by UV spectrophotometry at 260 nm (ND1000 Spectrophotometer; NanoDrop Technologies, Wilmington, USA). Real-time PCR was performed using the 7500 Real-time PCR System (Applied Biosystems, Foster City, USA) and SYBR® Green was used to detect fluorescence in PCR reaction, according to the manufacturer's instructions (Invitrogen, San Francisco, USA). The reaction conditions followed the protocols using primer pairs, forward, 5′-CACAGAAG GGACAGAAGT-3′ and reverse, 5′-TCGCCTTCATCTAC AGTC-3′) for amplification of *T. gondii*, as previously described (Homan et al., 2000; Wahab et al., 2010).

### Statistical analysis

The data were analyzed using GraphPad Prism 6.0 software package (GraphPad Software Inc., San Diego, USA). Differences among experimental and control groups were analyzed using One-way ANOVA with Tukey's multiple comparison post-test. Data were expressed as mean ± standard deviation (*SD*). The Kaplan-Meier method was applied to estimate the percentage of mice surviving at each time point after infection and survival curves were compared using the Log-rank Mantel-Cox test. Values of *P* < 0.05 were considered statistically significant.

## Results

### BMDM viability after treatment with ScLL or ArtinM

**Figure 1:**
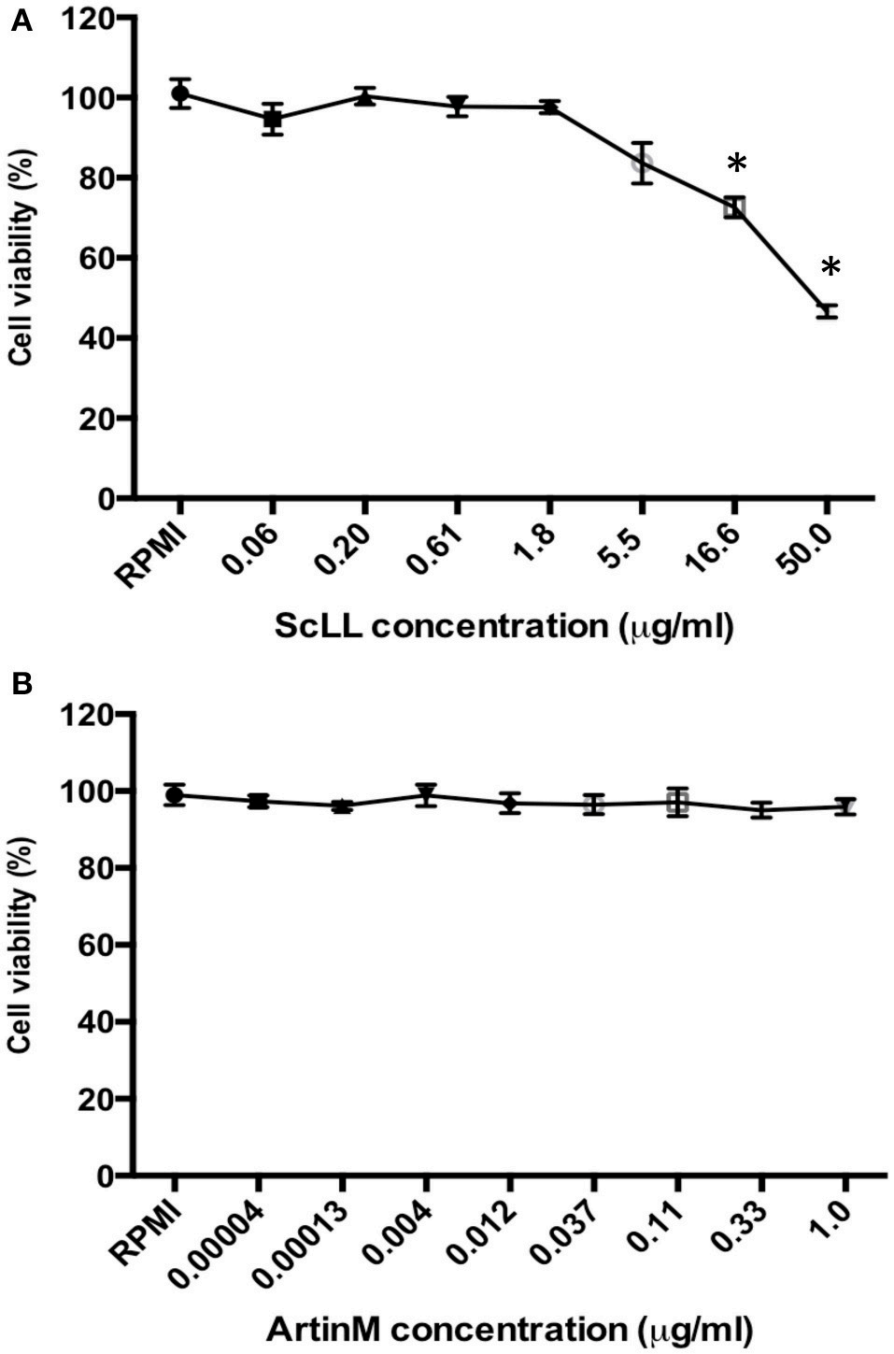
**Cell viability determined by MTT assay in murine bone marrow-derived macrophages (BMDMs), treated with ScLL (A)** or ArtinM **(B)** in several concentrations, ranging from 0.06 to 50 μg/ml or from 0.00004 to 1 μg/ml of ScLL or ArtinM, respectively. The cell suspensions were cultured in RPMI-1640 medium and the data are representative of one from three independent experiments. ^*^P < 0.05.

It was observed a dose-dependent cytotoxicity in BMDM treated with ScLL. These cells treated with higher concentration of ScLL (50 μg/mL) showed less than 50% of viability, whereas the total number of live cells was significantly increased when the ScLL concentrations were reduced, showing viability rates higher than 80% for doses equal or lower than 5.5 μg/mL the (Figure 1A). In contrast, for ArtinM, the viability rates were higher than 90% even at the higher concentration of 1 μg/mL (Figure 1B).

### Cytokines production in BMDM under ScLL or ArtinM treatment

**Figure 2:**
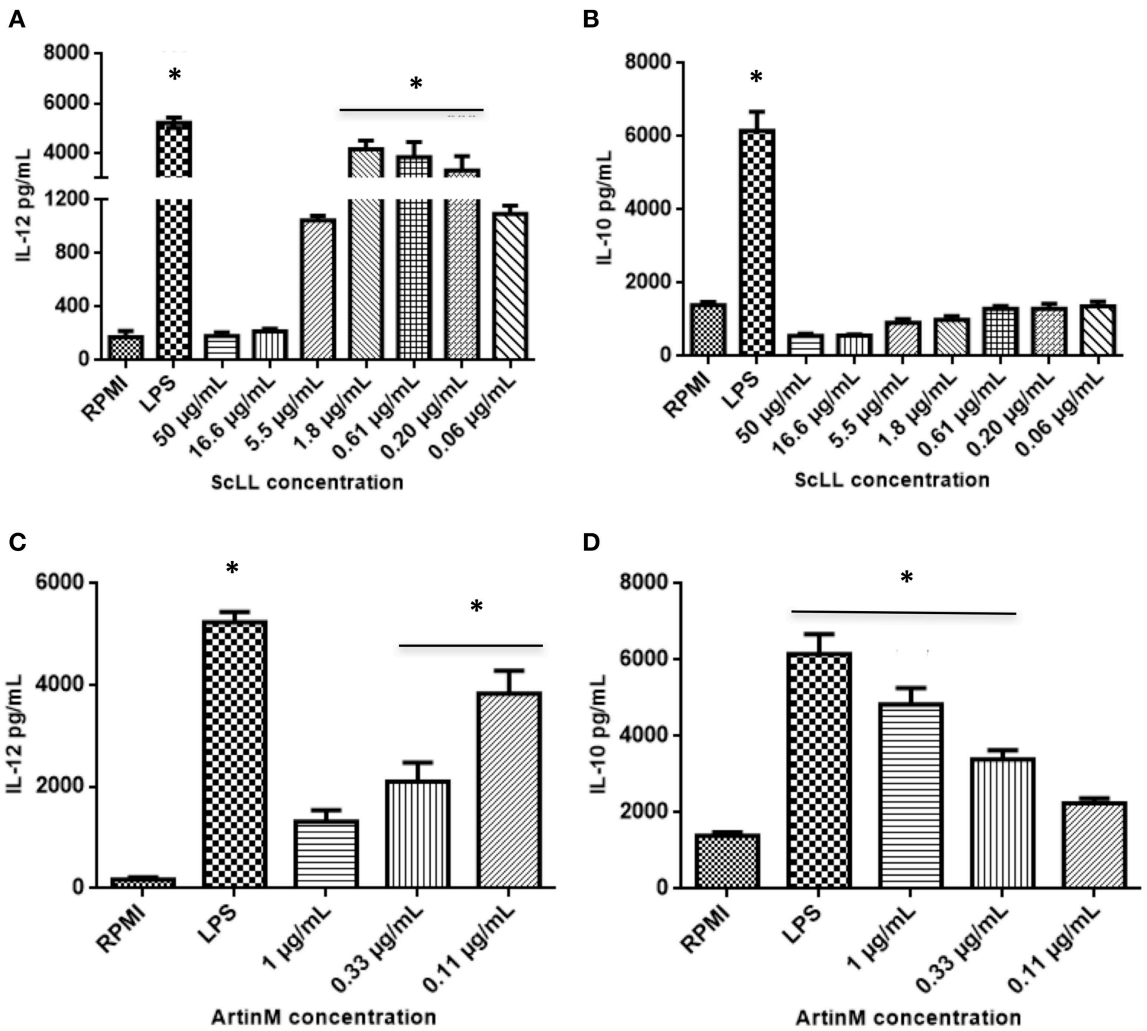
**Cytokine measurements in supernatants from bone marrow-derived macrophages (BMDMs) cultures after 48 h of stimulation with different concentrations of ScLL or ArtinM**. Medium alone (RPMI) or LPS (1 μg/mL) were included in all experiments, as negative and positive controls, respectively. Levels of IL-12 **(A)** and **(C)** and IL-10 **(B)** and **(D)** were determined by immunoenzymatic assay ELISA. Results are expressed as mean ± SD of cytokine levels in pg/mL. ^*^Statistically significant in relation to the control (RPMI-1640). (ANOVA and Tukey's multiple comparison post-test; *P* < 0.05).

ScLL and ArtinM were able to induce the cytokines production by BMDM (Figure 2). Cells treated with ScLL in concentrations of 1.8; 0.61; or 0.20 μg/mL, it was observed strong IL-12 production, in similar levels to that induced by LPS stimulus (Figure 2A). On the other hand, ScLL treatment was not able to induce IL-10 production in this cell suspension (Figure 2B). Regarding cells treated with ArtinM, it was found that this lectin was able to stimulate IL-12 production, and the concentration of 0.11 μg/mL induced the higher level of this cytokine, achieving values similar to the LPS stimulus (Figure 2C). In addition, ArtinM was able to induce IL-10 production in a dose-dependent manner, achieving the highest level at 1.0 μg/mL (Figure 2D).

### Nitrite production in BMDM treated with ScLL or ArtinM

**Figure 3:**
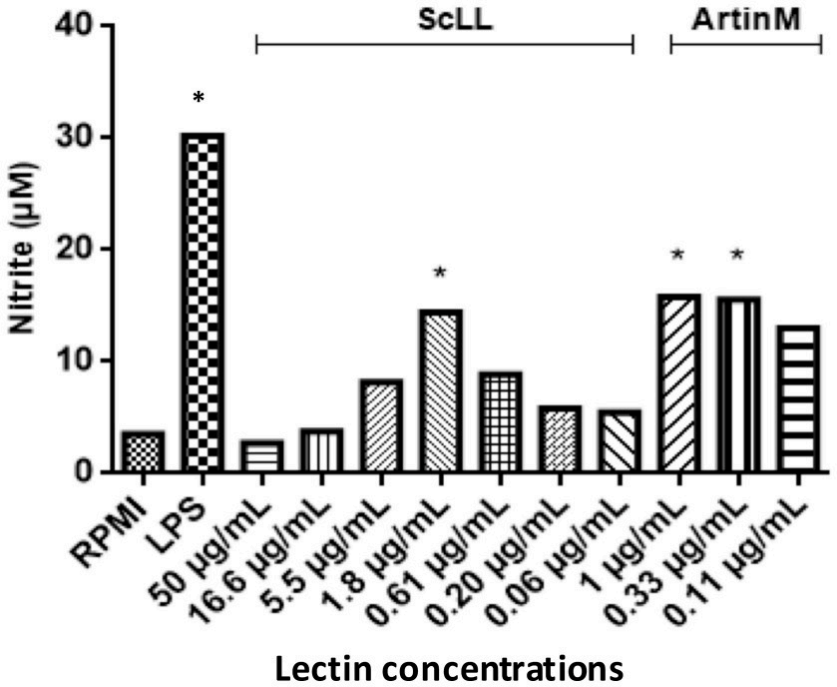
**Nitric oxide production in supernatants from bone marrow-derived macrophages (BMDMs) cultures after 48 h of stimulation with different concentrations of ScLL or ArtinM and LPS (1 μg/mL)**. Cells were stimulated with ScLL (50; 16.6; 5.5; 1.8; 0.61; 0.20; and 0.06 μg/mL) or ArtinM (1; 0.33 and 0.11 μg/mL). Nitrite concentration measured by Griess Reaction. The data are representative of one from three independent experiments in triplicate for each condition. ^*^Statistically significant in relation to the control (RPMI-1640). (ANOVA and Tukey's multiple comparison post-test; *P* < 0.05).

Nitrite concentration was measured in macrophages treated with ScLL or ArtinM, as an indicator of NO production. It was observed that both ScLL and ArtinM were able to stimulate NO production, being the higher level obtained in a concentration of 1.8 μg/mL for ScLL, while the concentrations of 1 and 0.33 μg/mL of ArtinM were equally able to induce higher levels of NO production (Figure 3).

### Cytokines production by *T. gondii*-infected C57BL/6 mice treated with ScLL and/or ArtinM lectins

**Figure 4:**
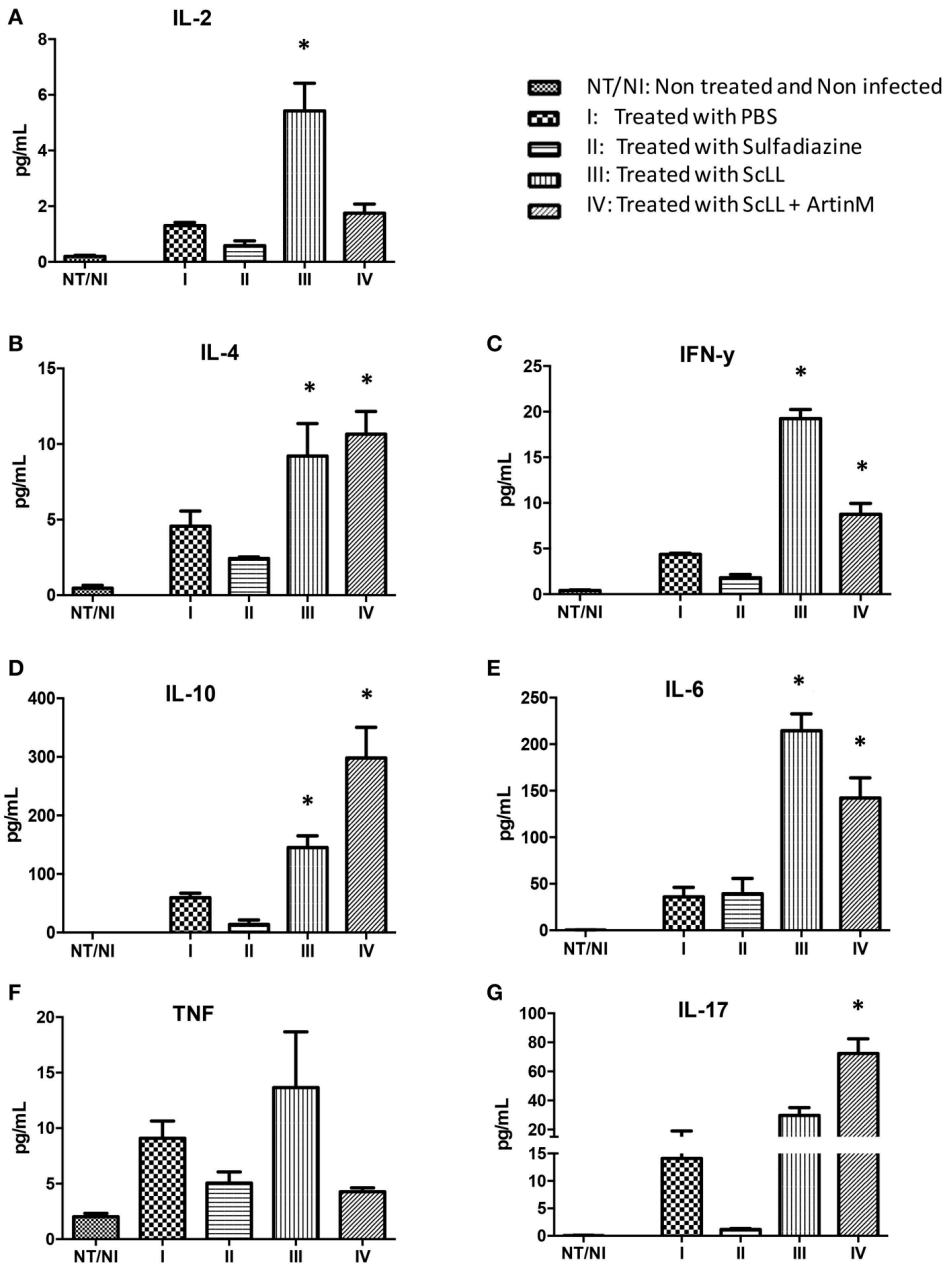
**Effects of sulfadiazine and lectin treatments in the levels of IL-2 (A)**, IL-4 **(B)**, IFN-γ **(C)**, IL-10 **(D)**, IL-6 **(E)**, TNF **(F)**, and IL-17 **(G)** cytokines in serum samples from C57BL/6 mice. Results are expressed as mean ± *SD* of the cytokine levels in pg/mL. Statistically significances were determined by comparison of the values obtained in the infected and treated groups with non-treated and non-infected mice. Statistical test was performed using One-way ANOVA and Tukey's multiple comparison post-test. ^*^Indicates significant differences (*P* < 0.05).

Given the efficiency of both lectins to stimulate cytokines production in BMDM, it was investigated whether ScLL and/or ArtinM treatments could be able to induce cytokines production by C57BL/6 infected mice. Analyses of cytokine levels showed that the treatment protocols with ScLL and/or ArtinM, consisting of six doses of 50 μg of ScLL, or six doses of 50 μg of ScLL plus 1 μg of ArtinM, increased significantly the concentrations of Th1, Th2, and Th17 cytokines when compared with non-treated or sulfadiazine-treated animals (Figure 4). However, a different profile concerning cytokines production was observed when the animals were treated with different lectin combinations. It was observed an induction higher of pro-inflammatory response in ScLL treated animals group compared to the ScLL plus ArtinM group. Indeed, IL-2, IFN-γ, and IL-6 production by infected and treated mice with ScLL was higher in comparison with ScLL plus ArtinM, as well as with sulfadiazine or non-treated infected controls (PBS) (*P* < 0.05) (Figures 4A,C,E). In contrast, ArtinM plus ScLL treatment induces higher levels of regulatory cytokines with a significant increase of IL-4, IL-10, and IL-17 in relation to others groups (*P* < 0.005) (Figures 4B,D,G). No significant difference in TNF production was observed among the treatments with lectins or sulfadiazine (Figure 4F). ScLL plus ArtinM demonstrated better balance of Th1/Th2 type of immunity response.

### Parasite burden in C57BL/6 mice infected by *T. gondii* and treat with ScLL and/or ArtinM lectins

**Figure 5:**
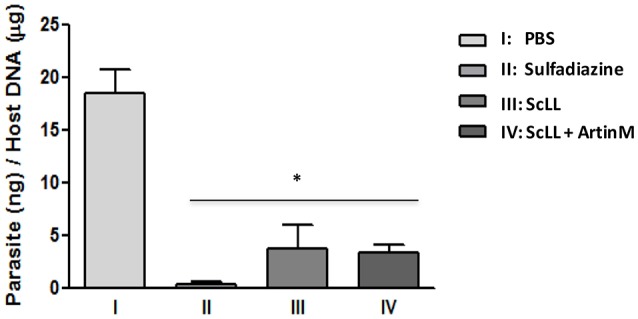
**Parasite burden in the brain tissues from treated mice after 30 days of infection with *T. gondii* ME-29 strain and analyzed by quantitative real-time PCR**. Statistical significances were calculated by comparison the parasitic DNA values among the treated and non-treated groups of mice. Statistical test was performed using One-way ANOVA and Tukey's multiple comparison post-test. ^*^Indicates significant differences (*P* < 0.05).

Parasite burden was examined in the brain tissues of the animals submitted to the different experimental procedures. As shown in Figure 5, it was observed that the treatments with either ScLL or ArtinM plus ScLL significantly reduced the parasite load in the central nervous system compared to infected but non-treated group (*P* < 0.05). In addition, it was not observed significant differences of *T. gondii*-DNA levels among the groups receiving lectins or sulfadiazine treatments, suggesting the ScLL and ArtinM could indeed reduce *T. gondii* burden to the central nervous system with the effectiveness comparable to the traditional chemotherapy.

### Survival rates of C57BL/6 mice infected by *T. gondii* and treated with ScLL and/or ArtinM lectins

**Figure 6:**
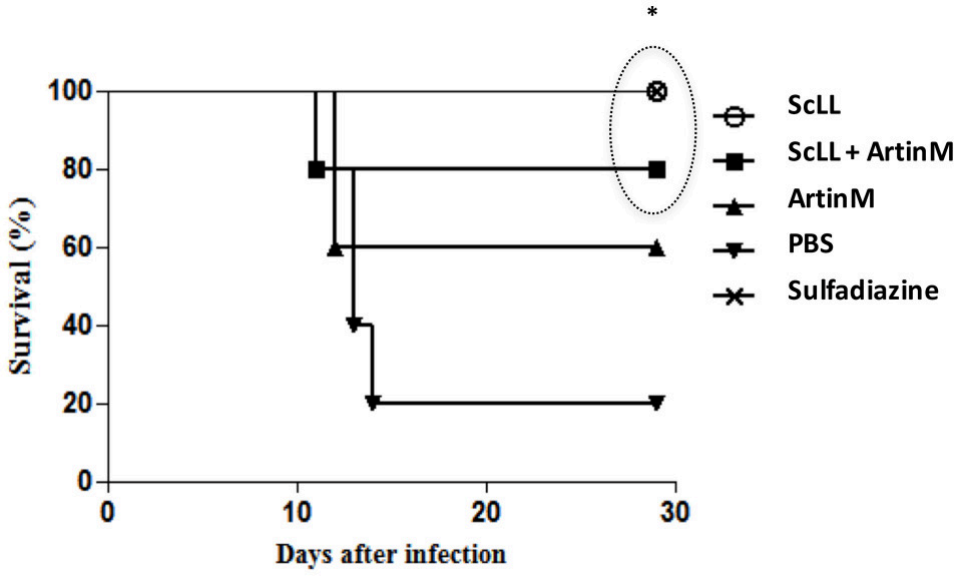
**Survival curve of C57BL/6 mice after infection with *T. gondii* ME49 strain**. Animals were treated with ScLL, ArtinM, ScLL plus ArtinM, or sulfadiazine during 6 days, one time a day. As control, animals were infected with the same strain, but treated with phosphate-buffered saline (PBS) only. The survival curves were compared using the Log-rank Mantel-Cox test. Values of *P* < 0.05 were considered statistically significant. ^*^Indicates significant differences (*P* < 0.05).

To certify whether the lectins treatments may interfere in the survival of C57BL/6 mice infected by *T. gondii*, when comparing to the groups of animals non-treated or submitted to chemotherapy, it was determined the survival rates 30-day post-infection. As shown in Figure 6, the animals submitted to the experimental treatment with lectins presented higher survival rates than the infected but non-treated mice. Interestingly, treatment with ScLL alone ensured a 100% survival of the animals, equal the survival rate observed to those animals submitted to the sulfadiazine treatment (Figure 6). Also, for the group treated with ScLL plus ArtinM, the survival rate was 80%, while the survival rate was 60% for the group of mice treated with ArtinM alone. The infected but non-treated group had the lowest survival rate of 20% (Figure 6), which was significantly lower than the rates determined to groups treated with ScLL, ScLL plus ArtinM or sulfadiazine (*P* < 0.05).

## Discussion

The strategies for toxoplasmosis treatment still show serious limitations, particularly due to the high degree of toxicity that has been associated with the current chemotherapy (Yeo et al., 2016). Also, the possibility to occur parasite drug resistance is an additional drawback to treat this disease based only on chemotherapy (Hui et al., 2015). Due the immunostimulatory effect of plant lectins, studies have been developed to implement of vaccination protocols using new agents to stimulate the immune system, as ScLL and ArtinM lectins extracted from plants (Cardoso et al., 2011, 2012; Souza et al., 2013). However, there is no previous study analyzing the therapeutic use of ScLL and ArtinM against *T. gondii* infection.

The association of sulfadiazine with pyrimethamine constitutes a protocol of treatment extensively used for toxoplasmosis, considering that it has been demonstrated to be highly effective against *T. gondii* tachyzoites, but it may cause severe hematological side effects, among other type of toxicities (Bosch-Driessen et al., 2002; Chêne and Thiébaut, 2009; Andrews et al., 2014). Previous *in vitro* and *in vivo* studies showed ScLL and ArtinM can be safely administered on murine models (Panunto-Castelo et al., 2001; Afonso-Cardoso et al., 2007; Cardoso et al., 2012). Furthermore, *in vivo* models demonstrated that ScLL presents no cytotoxic activity against J774 cells or peritoneal murine macrophages (Souza et al., 2005). In the present study, it was demonstrated that BMDM presented high rates of viability when treated with appropriate concentrations of ScLL and ArtinM lectins. Considering this framework, it is becoming very clear in the literature in last years that the beneficial effects of the lectins transcend the problems of the any potential side effect that could exist. Thus, the original use of lectins in vaccination protocols has been expanded to other applications. Hence, besides its use as therapeutic agents in infectious and parasitic diseases, lectins have been applied as new potential antineoplastic agents targeting apoptosis, autophagy, and anti-angiogenesis in preclinical or clinical trials for cancer therapeutics, and in neurodegenerative disorders, as well (Alegre-Maller et al., 2014; Bogoeva et al., 2014; Aminou et al., 2016; Barbosa-Lorenzi et al., 2016; Dar et al., 2016; Freitas et al., 2016; Ricci-Azevedo et al., 2016).

The role of cytokines in the immune response against *T. gondii* is well known and it has been extensively demonstrated that the regulation on Th1 pattern is necessary to control inflammatory damage on tissues (Gazzinelli et al., 1994, 1996; Lang et al., 2007). *T. gondii* control requires a fine-tuning induction of pro-inflammatory and anti-inflammatory cytokines, such as IL-12, IFN-γ, and IL-10 (Gazzinelli et al., 1991, 1996), but the tachyzoite stage of the parasite is able to subvert the immune response, being able, for instance, to blockade NF-κB nuclear translocation and actively interfere in the IL-12 production (Yarovinsky, 2014; Kim et al., 2016). Previous studies have been shown the ability of ScLL and ArtinM to induce the production of cytokines, when used as immunological adjuvants in vaccination protocols (Cardoso et al., 2011, 2012). In fact, when these lectins are used in murine models, they lead to the production of Th1, as well as Th2 cytokines, and these effects are important to avoid the deleterious consequences of unbalanced inflammatory cytokine production (Cardoso et al., 2011, 2012). In the present study, it was demonstrated that ScLL could stimulate *in vitro* IL-12 production by macrophages, but not IL-10, corresponding to an induction of Th1 pattern immune response, which is necessary, but not sufficient, to control *T. gondii* infection. However, for ArtinM-stimulated macrophages, it was observed the production of both IL-10 and IL-12. Interestingly, we also observed that the lowest ArtinM dose resulted in the highest IL-10 production.

Nitric oxide produced by macrophages has been demonstrated to be involved in anti-parasitic effects against various protozoa parasites, as well as in pathogenesis, including during *T. gondii* infection (Dincel and Atmaca, 2016). In the present study, it was observed that NO levels increased in macrophages stimulated *in vitro* with both ScLL and ArtinM. It has been described in murine models that, when the animals undergo the acute stage of infection by *T. gondii*, the NO production contributes to the tachyzoite-bradyzoite stage conversion (Ihara and Nishikawa, 2014). Our data showed that the non-toxic doses of ScLL as ArtinM are able to stimulate nitric oxide production by macrophages, which may be a relevant event to decrease *T. gondii* replication.

In the present study, it was established an intraperitoneal treatment protocol for toxoplasmosis, considering the results previously described (Cardoso et al., 2011, 2012). Once high concentration of ScLL showed cytotoxicity, it was evaluated different doses of this lectin in mice to determine the occurrence of side effects, as lethality test in mice. For this purpose, the animals received six doses of PBS or ScLL (50, 20, 10, and 5 μg). The animals were observed during 30 days for the pattern of morbidity (temperature, weight loss, mobility in the cage) and at the end of that period all the treated animals showed no alteration of their physical conditions (Data not shown).

Next, we investigated the capacity of the ScLL and ScLL plus ArtinM to increase cytokine production in infected animals. It was observed in the groups of animals treated with ScLL alone or with ScLL plus ArtinM increased levels of both pro-inflammatory and anti-inflammatory cytokines, showing differential, but complementary profiles. Immunostimulatory effects of ScLL and ArtinM leading to increase levels of IFN-γ, IL-6, TNF, IL-12, and IL-10 have been described for experimental designs set up in vaccination protocols in murine models (Afonso-Cardoso et al., 2007; Cardoso et al., 2011). Here, we are demonstrating for the first time the cytokine production profile, induced by *T. gondii* infected mice and treated with ScLL and ArtinM. Taken into account that it is necessary new treatment alternatives for toxoplasmosis, due to the limitations of the current protocols, as their toxic effect and development of parasites resistance (Antczak et al., 2016), the treatment exploring the immunostimulatory ability of lectins could be considered a rational alternative, since it is based on the induction of defense mechanisms of the host against *T. gondii* infection.

Following the cytokine analysis, it was investigated in the present study the parasite burden in brain tissues of treated mice, using quantitative PCR (qPCR) to detect specific *T. gondii* DNA. As expected, the highest parasite load was observed in non-treated mice. The parasite burden did not show significantly difference between mice treated with sulfadiazine or lectins, revealing the potential ability of lectins in parasite control. Sulfadiazine is effective for toxoplasmosis treatment, however, it has harmful effects to the organism. Currently, toxoplasmosis is still treated with the synergistic combination of sulfadiazine and pyrimethamine or triple sulfonamides, which cause bone marrow damage, in addition to the potential life-threatening allergic reactions. For these reasons, alternative drugs as treatment options, including azithromycin, clarithromycin, dapsone, and artemisinin, have been studied for toxoplasmosis treatment, but an extensive number of side effects still persists (Araujo et al., 1992a,b; Bosch-Driessen et al., 2002; D'Angelo et al., 2009; de Oliveira et al., 2009; Yan et al., 2013; Rostkowska et al., 2016). Thus, it is necessary to investigate alternative approaches to find efficient and well-tolerated therapeutic agents against *T. gondii* infection. In the current study, it was demonstrated that treatment protocols using ScLL or ArtinM plus ScLL were able to reduce parasite burden for C57BL/6 mice recently infected by ME49 strain of *T. gondii* without side effects.

It has been extensively described in the literature that C57BL/6 mice are highly susceptible to infection by *T. gondii*, as they are not able to efficiently control the acute phase of infection, presenting high rates of mortality (Sánchez et al., 2015). In the present study, it was observed a significant protective effect of ScLL and ArtinM treatments in mice orally infected by ME49 strain of the parasite. In fact, when compared to non-treated group of animals, high percentages of treated animals survived during the acute phase of *T. gondii* infection. Regarding the different experimental groups, C57BL/6 mice treated with ScLL alone or ScLL plus ArtinM presented survival rates of 100 and 80%, respectively, in contrast with non-treated animals, which presented significant lower survival rate.

Altogether, the results obtained in present study indicate important immunostimulatory effects of ScLL and ArtinM that was able to control *T. gondii* infection. Further studies will be necessary to evaluate different protocols to optimize the efficacy of ScLL and ArtinM lectins on this protozoan infection, particularly those carry out with different parasite genotypes.

## Author contributions

LS was involved in lectin preparation, cell culture, animal procedures, cytokine, and antibody assays, determination of brain parasite load for qPCR and preparation of the manuscript draft. ER participated in mouse infection and treatment procedures, cytokine and antibody assays. SS and MS were involved in inbred mouse maintenance, cytokine and antibody assays, and statistical analysis. FS was involved in the parasite maintenance in cell culture, *T. gondii* antigen preparation and lectin preparations. TM and JM were involved in the experimental design, data analysis, and revision of the manuscript. All authors read and approved the manuscript.

## Funding

This work was supported by Brazilian Research Agencies-CNPq (Procs.#311787/2013-4 and 456650/2013-0), FAPEMIG (Procs.#RED-00013-14 and #APQ-01313-14), and CAPES (Proc.#AUXPE-02450/09-7).

### Conflict of interest statement

The authors declare that the research was conducted in the absence of any commercial or financial relationships that could be construed as a potential conflict of interest.

## References

[B1] Adams Waldorf K. M. McAdams R. M. (2013). Influence of infection during pregnancy on fetal development. Reproduction 146, 151–162. 10.1530/REP-13-0232 23884862 PMC4060827

[B2] Afonso-Cardoso S. R. Rodrigues F. H. Gomes M. A. Silva A. G. Rocha A. Guimaraes A. H.. (2007). Protective effect of lectin from *Synadenium carinatum* on *Leishmania amazonensis* infection in BALB/c mice. Korean J. Parasitol. 45, 255–266. 10.3347/kjp.2007.45.4.255 18165707 PMC2532627

[B3] Alegre-Maller A. C. Mendonça F. C. da Silva T. A. Oliveira A. F. Freitas M. S. Hanna E. S.. (2014). Therapeutic administration of recombinant Paracoccin confers protection against *Paracoccidioides brasiliensis* infection: involvement of TLRs. PLoS Negl. Trop. Dis. 8:e3317. 10.1371/journal.pntd.0003317 25474158 PMC4256291

[B4] Aminou H. A. Alam-Eldin Y. H. Hashem H. A. (2016). Effect of *Nigella sativa* alcoholic extract and oil, as well as *Phaseolus vulgaris* (kidney bean) lectin on the ultrastructure of *Trichomonas vaginalis* trophozoites. J. Parasit. Dis. 40, 707–713. 10.1007/s12639-014-0564-x 27605771 PMC4996177

[B5] Andrews K. T. Fisher G. Skinner-Adams T. S. (2014). Drug repurposing and human parasitic protozoan diseases. Int. J. Parasitol. Drugs Resist. 4, 95–111. 10.1016/j.ijpddr.2014.02.002 25057459 PMC4095053

[B6] Antczak M. Dzitko K. Dlugońska H. (2016). Human toxoplasmosis–Searching for novel chemotherapeutics. Biomed. Pharmacother. 82, 677–684. 10.1016/j.biopha.2016.05.041 27470411

[B7] Araujo F. G. Lin T. Remington J. S. (1992a). Synergistic combination of azithromycin and sulfadiazine for treatment of toxoplasmosis in mice. Eur. J. Clin. Microbiol. Infect. Dis. 11, 71–73. 1314177 10.1007/BF01971278

[B8] Araujo F. G. Prokocimer P. Lin T. Remington J. S. (1992b). Activity of clarithromycin alone or in combination with other drugs for treatment of murine toxoplasmosis. Antimicrob. Agents Chemother. 36, 2454–2457. 1489188 10.1128/aac.36.11.2454 PMC284352

[B9] Bal A. Dhooria S. Agarwal R. Garg M. Das A. (2014). Multiple and atypical opportunistic infections in a HIV patient with Toxoplasma myocarditis. Cardiovasc. Pathol. 23, 358–362. 10.1016/j.carpath.2014.06.002 25060385

[B10] Barbosa-Lorenzi V. C. Cecilio N. T. de Almeida Buranello P. A. Pranchevicius M. C. Goldman M. H. Pereira-da-Silva G.. (2016). Recombinant ArtinM activates mast cells. BMC Immunol. 17:22. 10.1186/s12865-016-0161-0 27377926 PMC4932716

[B11] Blader I. J. Coleman B. I. Chen C. T. Gubbels M. J. (2015). Lytic cycle of *Toxoplasma gondii*: 15 years later. Annu. Rev. Microbiol. 69, 463–485. 10.1146/annurev-micro-091014-104100 26332089 PMC4659696

[B12] Bogoeva V. P. Petrova L. P. Trifonov A. A. (2014). New activity of a protein from *Canavalia ensiformis*. Sci. Pharm. 82, 825–834. 10.3797/scipharm.1404-09 26171327 PMC4475797

[B13] Bosch-Driessen L. H. Verbraak F. D. Suttorp-Schulten M. S. van Ruyven R. L. Klok A. M. Hoyng C. B.. (2002). A prospective, randomized trial of pyrimethamine and azithromycin vs pyrimethamine and sulfadiazine for the treatment of ocular toxoplasmosis. Am. J. Ophthalmol. 134, 34–40. 10.1016/S0002-9394(02)01537-4 12095805

[B14] Bradford M. M. (1976). A rapid and sensitive method for the quantitation of microgram quantities of protein utilizing the principle of protein-dye binding. Anal. Biochem. 72, 248–254. 942051 10.1016/0003-2697(76)90527-3

[B15] Bunn-Moreno M. M. Campos-Neto A. (1981). Lectin(s) extracted from seeds of *Artocarpus integrifolia* (jackfruit): potent and selective stimulator(s) of distinct human T and B cell functions. J. Immunol. 127, 427–429. 6972961

[B16] Cardoso M. R. Mota C. M. Ribeiro D. P. Noleto P. G. Andrade W. B. Souza M. A.. (2012). Adjuvant and immunostimulatory effects of a D-galactose-binding lectin from *Synadenium carinatum* latex (ScLL) in the mouse model of vaccination against neosporosis. Vet. Res. 43:76. 10.1186/1297-9716-43-76 23107170 PMC3583070

[B17] Cardoso M. R. Mota C. M. Ribeiro D. P. Santiago F. M. Carvalho J. V. Araujo E. C.. (2011). ArtinM, a D-mannose-binding lectin from *Artocarpus integrifolia*, plays a potent adjuvant and immunostimulatory role in immunization against *Neospora caninum*. Vaccine 29, 9183–9193. 10.1016/j.vaccine.2011.09.136 22001880

[B18] Chêne G. Thiébaut R. (2009). Options for clinical trials of pre and post-natal treatments for congenital toxoplasmosis. Mem. Inst. Oswaldo Cruz 104, 299–304. 10.1590/S0074-02762009000200025 19430657

[B19] D'Angelo J. G. Bordón C. Posner G. H. Yolken R. Jones-Brando L. (2009). Artemisinin derivatives inhibit *Toxoplasma gondii in vitro* at multiple steps in the lytic cycle. J. Antimicrob. Chemother. 63, 146–150. 10.1093/jac/dkn451 18988681 PMC2639242

[B20] Dar P. A. Singh L. R. Kamal M. A. Dar T. A. (2016). Unique medicinal properties of *Withania somnifera*: phytochemical constituents and protein pomponent. Curr. Pharm. Des. 22, 535–540. 10.2174/1381612822666151125001751 26601969

[B21] de Oliveira T. C. Silva D. A. Rostkowska C. Bela S. R. Ferro E. A. Magalhaes P. M. (2009). *Toxoplasma gondii*: effects of *Artemisia annua* L. on susceptibility to infection in experimental models *in vitro* and *in vivo*. Exp. Parasitol. 122, 233–241. 10.1016/j.exppara.2009.04.010 19389400

[B22] Dincel G. C. Atmaca H. T. (2016). Role of oxidative stress in the pathophysiology of *Toxoplasma gondii* infection. Int. J. Immunopathol. Pharmacol. 29, 226–240. 10.1177/0394632016638668 26966143 PMC5806720

[B23] Doliwa C. Xia D. Escotte-Binet S. Newsham E. L. Sanya J. S. Aubert D.. (2013). Identification of differentially expressed proteins in sulfadiazine resistant and sensitive strains of *Toxoplasma gondii* using difference-gel electrophoresis (DIGE). Int. J. Parasitol. Drug Resist. 3, 35–44. 10.1016/j.ijpddr.2012.12.002 24533291 PMC3862439

[B24] Dubey J. P. Lago E. G. Gennari S. M. Su C. Jones J. L. (2012). Toxoplasmosis in humans and animals in Brazil: high prevalence, high burden of disease, and epidemiology. Parasitology 139, 1375–1424. 10.1017/S0031182012000765 22776427

[B25] Dubey J. P. Lindsay D. S. Speer C. A. (1998). Structures of *Toxoplasma gondii* tachyzoites, bradyzoites, and sporozoites and biology and development of tissue cysts. Clin. Microbiol. Rev. 11, 267–299. 9564564 10.1128/cmr.11.2.267 PMC106833

[B26] Dupont C. D. Christian D. A. Hunter C. A. (2012). Immune response and immunopathology during toxoplasmosis. Semin. Immunopathol. 34, 793–813. 10.1007/s00281-012-0339-3 22955326 PMC3498595

[B27] Elsheikha H. M. (2008). Safer food for pregnant women: practices and risks. Public Health 122, 1407–1409. 10.1016/j.puhe.2008.06.002 18778840

[B28] Enzensberger W. Helm E. B. Hopp G. Stille W. Fischer P. A. (1985). Toxoplasmosis encephalitis in patients with AIDS. Dtsch. Med. Wochenschr. 110, 83–87. 3967592 10.1055/s-2008-1068778

[B29] Ferro E. A. Silva D. A. Bevilacqua E. Mineo J. R. (2002). Effect of *Toxoplasma gondii* infection kinetics on trophoblast cell population in *Calomys callosus*, a model of congenital toxoplasmosis. Infect. Immun. 70, 7089–7094. 10.1128/IAI.70.12.7089-7094.2002 12438390 PMC133059

[B30] Freitas M. S. Oliveira A. F. da Silva T. A. Fernandes F. F. Gonçales R. A. Almeida F.. (2016). Paracoccin induces M1 polarization of macrophages via interaction with TLR4. Front. Microbiol. 7:1003. 10.3389/fmicb.2016.01003 27458431 PMC4932198

[B31] Gazzinelli R. T. Hakim F. T. Hieny S. Shearer G. M. Sher A. (1991). Synergistic role of CD4+ and CD8+ T lymphocytes in IFN-gamma production and protective immunity induced by an attenuated *Toxoplasma gondii* vaccine. J. Immunol. 146, 286–292. 1670604

[B32] Gazzinelli R. T. Wysocka M. Hayashi S. Denkers E. Y. Hieny S. Caspar P.. (1994). Parasite-induced IL-12 stimulates early IFN-gamma synthesis and resistance during acute infection with *Toxoplasma gondii*. J. Immunol. 153, 2533–2543. 7915739

[B33] Gazzinelli R. T. Wysocka M. Hieny S. Scharton-Kersten T. Cheever A. Kuhn R.. (1996). In the absence of endogenous IL-10, mice acutely infected with *Toxoplasma gondii* succumb to a lethal immune response dependent on CD4+ T cells and accompanied by overproduction of IL-12, IFN-gamma and TNF-alpha. J. Immunol. 157, 798–805. 8752931

[B34] Green L. C. Wagner D. A. Glogowski J. Skipper P. L. Wishnok J. S. Tannenbaum S. R. (1982). Analysis of nitrate, nitrite, and [15N]nitrate in biological fluids. Anal. Biochem. 126, 131–138. 7181105 10.1016/0003-2697(82)90118-x

[B35] Homan W. L. Vercammen M. De Braekeleer J. Verschueren H. (2000). Identification of a 200- to 300-fold repetitive 529 bp DNA fragment in *Toxoplasma gondii*, and its use for diagnostic and quantitative PCR. Int. J. Parasitol. 30, 69–75. 10.1016/S0020-7519(99)00170-8 10675747

[B36] Hui R. El Bakkouri M. Sibley L. D. (2015). Designing selective inhibitors for calcium-dependent protein kinases in apicomplexans. Trends Pharmacol. Sci. 36, 452–460. 10.1016/j.tips.2015.04.011 26002073 PMC4485940

[B37] Ihara F. Nishikawa Y. (2014). Starvation of low-density lipoprotein-derived cholesterol induces bradyzoite conversion in *Toxoplasma gondii*. Parasit. Vectors 7:248. 10.1186/1756-3305-7-248 24885547 PMC4046157

[B38] Jones J. L. Lopez A. Wilson M. Schulkin J. Gibbs R. (2001). Congenital toxoplasmosis: a review. Obstet. Gynecol. Surv. 56, 296–305. 11333376 10.1097/00006254-200105000-00025

[B39] Kaye A. (2011). Toxoplasmosis: diagnosis, treatment, and prevention in congenitally exposed infants. J. Pediatr. Health Care 25, 355–364. 10.1016/j.pedhc.2010.04.008 22018426

[B40] Kim E. W. Nadipuram S. M. Tetlow A. L. Barshop W. D. Liu P. T. Wohlschlegel J. A.. (2016). The rhoptry pseudokinase ROP54 modulates *Toxoplasma gondii* virulence and host GBP2 loading. mSphere 1, e00045–e00016. 10.1128/mSphere.00045-16 27303719 PMC4863586

[B41] Laemmli U. K. (1970). Cleavage of structural proteins during the assembly of the head of bacteriophage T4. Nature 227, 680–685. 5432063 10.1038/227680a0

[B42] Lang C. Gross U. Lüder C. G. (2007). Subversion of innate and adaptive immune responses by *Toxoplasma gondii*. Parasitol. Res. 100, 191–203. 10.1007/s00436-006-0306-9 17024357

[B43] Lopes A. P. Dubey J. P. Dardé M. L. Cardoso L. (2014). Epidemiological review of *Toxoplasma gondii* infection in humans and animals in Portugal. Parasitology 141, 1699–1708. 10.1017/S0031182014001413 25215422

[B44] Marim F. M. Silveira T. N. Lima D. S. Jr. Zamboni D. S. (2010). A method for generation of bone marrow-derived macrophages from cryopreserved mouse bone marrow cells. PLoS ONE 5:e15263. 10.1371/journal.pone.0015263 21179419 PMC3003694

[B45] Meneceur P. Bouldouyre M.-A. Aubert D. Villena I. Menotti J. Sauvage V.. (2008). *In vitro* susceptibility of various genotypic strains of *Toxoplasma gondii* to pyrimethamine, sulfadiazine, and atovaquone. Antimicrob. Agents Chemother. 52, 1269–1277. 10.1128/AAC.01203-07 18212105 PMC2292506

[B46] Miller S. A. Dykes D. D. Polesky H. F. (1988). A simple salting out procedure for extracting DNA from human nucleated cells. Nucleic Acids Res. 16, 1215. 3344216 10.1093/nar/16.3.1215 PMC334765

[B47] Montoya J. G. Liesenfeld O. (2004). Toxoplasmosis. Lancet 363, 1965–1976. 10.1016/S0140-6736(04)16412-X 15194258

[B48] Montoya J. G. Remington J. S. (2008). Management of *Toxoplasma gondii* infection during pregnancy. Clin. Infect. Dis. 47, 554–566. 10.1086/590149 18624630

[B49] Mori T. Kato J. Okamoto S. (2011). Pancytopenia due to pyrimethamine triggered by transplant-associated microangiopathy after allogeneic bone marrow transplantation. J. Infect. Chemother. 17, 866–867. 10.1007/s10156-011-0266-6 21674200

[B50] Mosmann T. (1983). Rapid colorimetric assay for cellular growth and survival: application to proliferation and cytotoxicity assays. J. Immunol. Methods 65, 55–63. 6606682 10.1016/0022-1759(83)90303-4

[B51] Oliveira C. B. Meurer Y. S. Andrade J. M. Costa M. E. Andrade M. M. Silva L. A.. (2016). Pathogenicity and phenotypic sulfadiazine resistance of *Toxoplasma gondii* isolates obtained from livestock in northeastern Brazil. Mem. Inst. Oswaldo Cruz. 111, 391–398. 10.1590/0074-02760150459 27276184 PMC4909038

[B52] Panunto-Castelo A. Souza M. A. Roque-Barreira M. C. Silva J. S. (2001). KM(+), a lectin from *Artocarpus integrifolia*, induces IL-12 p40 production by macrophages and switches from type 2 to type 1 cell-mediated immunity against *Leishmania major* antigens, resulting in BALB/c mice resistance to infection. Glycobiology 11, 1035–1042. 10.1093/glycob/11.12.1035 11805076

[B53] Ricci-Azevedo R. Oliveira A. F. Conrado M. C. Carvalho F. C. Roque-Barreira M. C. (2016). Neutrophils contribute to the protection conferred by ArtinM against intracellular pathogens: a study on *Leishmania major*. PLoS Negl. Trop. Dis. 10:e0004609. 10.1371/journal.pntd.0004609 27058234 PMC4825989

[B54] Rodriguez J. B. Szajnman S. H. (2012). New antibacterials for the treatment of toxoplasmosis: a patent review. Expert Opin. Ther. Pat. 22, 311–333. 10.1517/13543776.2012.668886 22404108

[B55] Roque-Barreira M. C. Praz F. Halbwachs-Mecarelli L. Greene L. J. Campos-Neto A. (1986). IgA-affinity purification and characterization of the lectin jacalin. Braz. J. Med. Biol. Res. 19, 149–157. 3828569

[B56] Rostkowska C. Mota M. C. Oliveira T. C. Santiago F. M. Oliveira L. A. Korndorfer G. H. (2016). Si-accumulation in *Artemisia annua* glandular trichomes increases artemisinin concentration, but does not interfere in the impairment of *Toxoplasma gondii* growth. Front Plant Sci. 7:1430 10.3389/fpls.2016.01430 27721819 PMC5033981

[B57] Samra N. A. McCrindle C. M. E. Penzhorn B. L. Cenci-Goga B. (2007). Seroprevalence of toxoplasmosis in sheep in South Africa. J. S. Afr. Vet. Assoc. 78, 116–120. 10.4102/jsava.v78i3.301 18237032

[B58] Sánchez V. R. Fenoy I. M. Picchio M. S. Soto A. S. Arcon N. Goldman A.. (2015). Homologous prime-boost strategy with TgPI-1 improves the immune response and protects highly susceptible mice against chronic *Toxoplasma gondii* infection. Acta Trop. 150, 159–165. 10.1016/j.actatropica.2015.07.013 26200784

[B59] Souza M. A. Cardoso C. R. B. Silva A. G. Silva E. G. Andrade L. R. Pena J. D. O. (2005). Isolation and partial characterization of a D-galactose-binding lectin from the latex of *Synadenium carinatum*. Braz. Arch. Biol. Technol. 48, 705–716. 10.1590/S1516-89132005000600005

[B60] Souza M. A. Carvalho F. C. Ruas L. P. Ricci-Azevedo R. Roque-Barreira M. C. (2013). The immunomodulatory effect of plant lectins: a review with emphasis on ArtinM properties. Glycoconj. J. 30, 641–657. 10.1007/s10719-012-9464-4 23299509 PMC3769584

[B61] Teixeira C. R. Cavassani K. A. Gomes R. B. Teixeira M. J. Roque-Barreira M. C. Cavada B. S.. (2006). Potential of KM+ lectin in immunization against *Leishmania amazonensis* infection. Vaccine 24, 3001–3008. 10.1016/j.vaccine.2005.11 16455170

[B62] Tenter A. M. Heckeroth A. R. Weiss L. M. (2000). *Toxoplasma gondii*: from animals to humans. Int. J. Parasitol. 30, 1217–1258. 10.1016/S0020-7519(00)00124-7 11113252 PMC3109627

[B63] Toledo K. A. Scwartz C. Oliveira A. F. Conrado M. C. Bernardes E. S. Fernandes L. C.. (2009). Neutrophil activation induced by ArtinM: release of inflammatory mediators and enhancement of effector functions. Immunol. Lett. 123, 14–20 10.1016/j.imlet.2009.01.009 19428547

[B64] Vijayalaxmi K. K. Vishalakshi M. (2000). Evaluation of the genotoxic effects of pyrimethamine, an antimalarial drug, in the *in vivo* mouse. Teratog. Carcinog. Mutagen 20, 65–71. 10.1002/(SICI)1520-6866(2000)20:2<65::AID-TCM2>3.0.CO;2-K 10679750

[B65] Wahab T. Edvinsson B. Palm D. Lindh J. (2010). Comparison of the AF146527 and B1 repeated elements, two real-time PCR targets used for detection of *Toxoplasma gondii*. J. Clin. Microbiol. 48, 591–592. 10.1128/JCM.01113-09 19940050 PMC2815584

[B66] Yan J. Huang B. Liu G. Wu B. Huang S. Zheng H.. (2013). Meta-analysis of prevention and treatment of toxoplasmic encephalitis in HIV-infected patients. Acta Trop. 127, 236–244. 10.1016/j.actatropica.2013.05.006 23707647

[B67] Yarovinsky F. (2014). Innate immunity to *Toxoplasma gondii* infection. Nat. Rev. Immunol. 14, 109–121. 10.1038/nri3598 24457485

[B68] Yeo S. J. Jin C. Kim S. Park H. (2016). *In vitro* and *in vivo* effects of nitrofurantoin on experimental toxoplasmosis. Korean J. Parasitol. 54, 155–161. 10.3347/kjp.2016.54.2.155 27180573 PMC4870977

